# Cannot Target What Cannot Be Seen: Molecular Imaging of Cancer Stem Cells

**DOI:** 10.3390/ijms24021524

**Published:** 2023-01-12

**Authors:** Loredana G. Marcu, Leyla Moghaddasi, Eva Bezak

**Affiliations:** 1 Faculty of Informatics and Science, University of Oradea, 1 Universitatii Str., 410087 Oradea, Romania; 2 Cancer Research Institute, University of South Australia, Adelaide, SA 5001, Australia; 3 Northern Sydney Cancer Centre, Royal North Shore Hospital, St. Leonards, NSW 2065, Australia; 4 School of Physical Sciences, University of Adelaide, Adelaide, SA 5005, Australia

**Keywords:** functional imaging, positron emission tomography (PET), single photon emission tomography (SPECT), magnetic resonance imaging (MRI), personalised therapy

## Abstract

Cancer stem cells are known to play a key role in tumour development, proliferation, and metastases. Their unique properties confer resistance to therapy, often leading to treatment failure. It is believed that research into the identification, targeting, and eradication of these cells can revolutionise oncological treatment. Based on the principle that what cannot be seen, cannot be targeted, a primary step in cancer management is the identification of these cells. The current review aims to encompass the state-of-the-art functional imaging techniques that enable the identification of cancer stem cells via various pathways and mechanisms. The paper presents in vivo molecular techniques that are currently available or await clinical implementation. Challenges and future prospects are highlighted to open new research avenues in cancer stem cell imaging.

## 1. The Necessity for Research into the Imaging of Cancer Stem Cells

The path followed by current molecular oncological research leads towards personalised therapy, which is the goal of today’s medicine. Substantial research is therefore oriented towards tumour-specific properties to assist with the development of targeted therapies. Tumour-specific factors such as hypoxia, angiogenesis, and proliferation are presently among the most researched properties, leading to a lot of effort to identify and target hypoxic cells, epidermal growth factor receptor (EGFR)-related pathways, and vascular endothelial growth factor receptor (VEGF)-related pathways. Nevertheless, cancer cell heterogeneity continues to pose challenges through tumour aggressiveness, resistance to treatment, and cancer dissemination to distant sites.

Since the first evidence towards the existence of a rare population of stem-like cancer cells, the field of cancer stem cells (CSC) has become one of the most thought-provoking areas of current oncological research. Yet, not enough attention is given to the translational aspect of this research, despite the ample evidence showing their key role in cancer development, proliferation, and dissemination. Owing to their unique tumour-promoting properties, cancer stem cells are most often the culprits of treatment failure in oncology [1,2]. The management of cancer stem cells must be conducted through specific targeting and eradication. Since one cannot target what one cannot see, an important aspect of research is the identification of CSCs. Due to their characteristic cell surface expression profile, CSCs can be isolated from other tumour cells, enabling the development of specific CSC markers to serve as imaging tools.

Recently, a number of techniques have been developed to support the molecular imaging of cancer stem cells. While some research is focused on the in vivo identification of CSCs via functional imaging through positron emission tomography (PET), single photon emission tomography (SPECT), or magnetic resonance imaging (MRI) [3], others have developed in vitro techniques for the identification of circulating cancer stem cells using liquid biopsies to identify the metastatic potential of primary tumours [4]. The field of functional imaging is continuously increasing with new radionuclides that have an affinity towards CSCs.

Imaging and targeting cancer stem cells could be the next step towards personalised therapy in oncology. In view of the above, the current review aims to compile the state-of-the-art in in vivo molecular imaging of cancer stem cells, identifying the opportunities for clinical translation as well as the challenges the research community is still facing.

## 2. The Multiple Facets of Cancer Stem Cells and Their Role in Tumour Development and Progression

While a few decades ago the mere existence of cancer stem cells was still questionable, the contribution of evidence-based medicine to today’s knowledge confirms the presence of primitive, undifferentiated cancer cells within most neoplasms, that exhibit similar biological properties, signalling pathways, and expression profiles to their normal complements [5]. These cancer cells with stem-like properties are pluripotent, being able to maintain an undifferentiated state and self-renew but also to differentiate into all heterogeneous lineages of the original tumour [6].

Despite their presence in very small fractions within most tumours, cancer stem cells were shown to be highly potent in dictating tumour growth, proliferation, and distant metastases, being responsible for tumour recurrence, treatment resistance, and failure [1] (Table 1).

Among the commonly shared properties between normal and cancer stem cells is their self-renewing ability, allowing for extensive proliferation and self-preservation. Nevertheless, proliferation is well-controlled in normal cells, whereas tumorigenic cells lack such a regulatory effect, leading to overpopulation of CSCs due to symmetrical division, and thus uncontrolled tumour growth. The capability to generate a lineage of differentiated progenies is another feature common to both normal and cancer cells. Differentiation of normal stem cells is mostly dictated by the microenvironment and the needs of specific organs to retain a homeostatic state (such as wound healing) [7]. On the other hand, the cell differentiation characteristic to CSCs is greatly dysregulated, resulting in a highly heterogeneous and phenotypically different cell population that is difficult to manage clinically [8]. Another distinct characteristic between normal and cancer stem cells is the preservation of genomic integrity. While this is a critical property of normal stem cells, tumour cells acquire a genetic mutation for their further progress and in order to adapt to various microenvironmental factors [9].

Although normal and cancer stem cells originating from the same organ can express similar surface receptors, the range of identified markers to date is limited [9]. In normal stem cells, the pattern of cell marker expression in a specific organ from different hosts is relatively stable, which is not the case for CSC markers, being suggestive of cancer stem cell plasticity.

The complexity of the tumour microenvironment also impacts the behaviour of cancer stem cells. Studies revealed a close association between CSCs and the immune environment, to an extent that CSCs often evade the immune response through the expression of immunosuppressive mediators [10]. Another proven characteristic of CSCs is their preference to dwell in very specific and often hostile microenvironmental niches in order to maintain their status [11,12]. These niches preserve the main properties of CSCs, including their plasticity, metastatic potential, and evasion of the immune system [13]. Owing to the specificity of the cancer microenvironment, CSC niches have been identified in a large number of malignancies stimulating research into cancer-specific characteristics in view of personalised therapies [14]. In certain tumours, CSCs were found to reside in perivascular niches, experiencing radical changes in their microenvironment through bouts of hypoxia and reoxygenation [15]. The hypoxic niches allow CSCs to prolong their cycle duration, thus imposing a very slow turnover, with hypoxia being a vital factor for the preservation of stem-like properties. An important player in the niche mechanisms is the hypoxia inducible factor (HIF) involved in cell survival under hypoxia, CSC proliferation, and in the activation of the angiogenic switch during cancer development [14]. The increased expression of HIF was found in hypoxic niches that showed the presence of CSCs [16]. This fact impacts tumour control, given that HIFs contribute towards treatment resistance through adaptation to hypoxic settings [17].

Suboptimal conditions within the tumour microenvironment and epigenetic modifications can also trigger cellular dormancy, which is a state that renders CSCs to reside in a quiescent phase, outside the cell cycle [18]. While they stop proliferating, dormant cells retain their viability and can potentially be recruited into the cell cycle to sustain cancer growth and proliferation [2]. There is evidence that even differentiated cells residing in a dormant phase can contribute to treatment resistance through de-differentiation that provides them with stem-like properties [19].

As a response to treatment-induced effects, CSCs are capable of efficient DNA repair [11], accelerated repopulation [20], and dynamic transitions between a quiescent and a migratory state [21], as also mentioned above. Radiotherapy, particularly fractionated irradiation, is shown to trigger plasticity, whereby non-stem cancer cells undergo de-differentiation towards stem-like phenotypes by induced epithelial-mesenchymal transition, thus increasing the pool of CSCs and supporting metastatic growth [22,23,24]. The pool of cancer stem cells can also increase through the various repopulation mechanisms displayed by stem-like cells. The common asymmetrical cellular division that results in a stem-like and differentiated cell is often converted into a symmetrical division that creates two stem-like cancer cells; this is one of the most powerful properties leading to accelerated cancer growth [25,26].

Research on the origins and properties of CSCs led to the identification of stem-like cancer cells via specific biomarkers in most tumour types, both solid and liquid. The analysis of CSC marker expressions across different human cell lines revealed notable variations among the fractions of CSC present in histopathologically different tumours. Consequently, CSC percentages in cell lines range from as low as 0.4% in acute myeloid leukemia to 82.7% found in colon cancers [27]. While the quantitative data reporting can be argumentative due to various markers and techniques used in quantifying the percentage of CSCs present in different tumours, noteworthy differences in CSC proportions were reported among head and neck cell lines, ranging from 1.7% to 13.5% [28]. This cell line dependence was also confirmed by other studies emphasising the need for CSC identification on an individual basis [29]. CSC markers also vary in their expression between cell lines, thus offering different information regarding the number of cells that test positive for a specific marker [30], an observation that can further bias the quantification of stem-like cells.

The in vivo scenario of CSC quantification is somewhat different, as most studies consistently report very small proportions of stem-like cancer cells in primary tumours (<1%). Their identification is facilitated by a large number of exclusive markers, most often used in the form of cell surface markers via a cluster of differentiation (CD), or specific enzymes such as aldehyde dehydrogenase (ALDH). There is experimental evidence showing that the percentage of CSCs in prostate cancers is below 0.1%, in breast cancer ranges between 0.1% and 1%, and pancreatic cancers contain 1–3% [31], whereas in ovarian cancers their frequency is very low (<0.04%) though with large variations among patients [32]. Undifferentiated tumours, as probably expected, were observed to exhibit higher percentages of CSCs [33]. Nevertheless, these fractions of CSCs in most cancers are probably underestimated owing to various factors such as marker specificity, differences in growth factors and receptors among tumours, and also the extent of immune recognition [34].

**Table 1:** Properties of cancer stem cells revealed by experimental studies.

Experiment-Based Properties of Cancer Stem Cells		References
Cellular and cell cycle-related properties	Long-lived (immortal)	[11]
	Can create all heterogeneous lineages of the original tumour	[6]
	Able to divide by symmetrical division	[26]
	Commonly dwell in microenvironmental niches within the tumour	[35]
	Undergo cell recruitment (from their niche into the mitotic cycle)	[2]
	Can present both a stationary state (quiescent) as well as a migratory state (invasive)	[21]
	Have different phenotypes that dictate their cellular behaviour	[36]
Radiobiological and treatment-related properties	Exhibit enhanced DNA repair compared to their non-stem counterparts	[11]
	Demonstrate higher treatment resistance than non-stem cancer cells	[11]
	Capable of tumour repopulation during treatment	[37]
	Can induce shortening of cell cycle duration in response to cell loss	[20,38]
	Undergo drastic changes in perivascular niches triggering bouts of hypoxia and reoxygenation	[15]
	Characterised by cellular plasticity (dynamic transformation from CSC into non-stem cancer cell and vice versa)	[39]
	Display altered cellular kinetics during fractionated radiotherapy	[40,41]
	Evade the immune response	[10]

The diversity in the phenotypic heterogeneity of solid tumours is known to be propelled by the variety of cell types, where CSCs play a critical role. Recent investigations have demonstrated that even CSC subpopulations present a phenotypical diversity of cancer stem cells, conferring different properties to the tumour as a whole [41,42]. Consequently, experimental data indicated that it is not only the fraction of CSCs within a tumour that dictates its fate but also the CSC phenotype [36,41,42]. The best example in view of the above statement is head and neck cancer, which shows a correlation between the biological behaviour/response to treatment and HPV status. Studies showed that HPV-positive tumours, which are known for their superior response to radio-chemotherapy compared to HPV-negative ones, often present with higher percentages of cancer stem cells than their HPV-negative counterparts, suggesting that the phenotype of CSCs is an essential factor to be considered when targeting cancer stem cells [36,41].

Another category of cancer cells that requires identification is the circulating tumour cell (CTC) population, responsible for tumour spread and the initiation of metastatic disease. CTCs are epithelial malignant cells separated from the primary tumour, which underwent the epithelial-mesenchymal transition (EMT), and through intravasation into the blood stream they reached distant anatomic sites in order to extravasate and to form micrometastases [43]. The composition of the CTC population is highly heterogenous, consisting of subpopulations of various phenotypes, including cells with stem-like properties [43]. Several studies found correlations between the amount and type of CTC identified in the blood and clinical outcome of cancer patients, showing that the detection of CTCs with stem-like properties could provide prognostic and predictive information alike, which would assist with treatment adaptation and personalisation [44,45].

All the above properties justify the need for the identification of cancer stem cells. Significant work has been done in the field of biomarkers that allow the labelling of stem-like cells, implicitly leading to the prospect of CSC identification in vitro and in vivo alike.

## 3. Current Biomarkers Specific for Cancer Stem Cells

The main characteristics of CSCs, differing them from normal healthy cells, have been described above. These characteristics can be, in turn, utilised in their identification. For example, the fact, that CSCs have modified their cellular surface in order to change their sensitivity to the regulating mechanisms of the host normal tissues [46], can be exploited by imaging. In other words, while changes in the CSCs’ membrane (e.g., in proteins, enzymes, cell surface receptors) play an important role in the process of malignisation, they also represent a point of differentiation between the normal and the malignant cells, and therefore can offer solutions/targets for cancer cell identification and therapy. These changes can include structural changes of proteins and surface receptors, different (increased^+^/decreased^−^) receptor expressions, and the presence of new surface molecules [46], i.e., cancer-specific surface characteristics that can be utilised in imaging as well as in targeted radionuclide therapy. For example, while mucin proteins are present on the most cells’ surfaces, changes in their glycosylation pattern in epithelial tumour tissues make them specific/distinctive of these cancer tissues. In regard to cancer stem cells, there is evidence that their surface markers can be different in some cases, but are often also very similar, if not the same, as embryonic stem, adult stem, and even normal tissue cell surface markers [47]. This is sometimes used to hypothesise that the CSCs have derived from heathy stem cells through a series of mutations. However, in the case of CSCs, the expression of surface markers (a single surface marker or several in combination) is up- or down-regulated as compared to normal cells.

There are several classes of receptors or cell surface markers that are characteristic of stem cells, and only some examples will be provided here (see Table 2). A very diverse series of membrane proteins, known as a cluster of differentiation (CD) markers or CD antigens, are a group of surface glycoproteins that provides an interface between a cell and other cells [48]. As mentioned above, many of these are up-regulated and sometimes down-regulated in CSCs. At present, the evidence is coming mostly from pre-clinical studies and their presence still needs to be validated conclusively [47]. The most frequently reported CSC surface marker is CD133: a five-transmembrane domain glycoprotein that localises to membrane protrusions and serves to properly maintain the lipid structure of the plasma membrane. This has been reported in many solid cancers including those of the lung, pancreas, brain, colon, ovaries, or hemopoietic system [49,50,51,52,53,54,55]. CD90 was reported as a marker for CSCs in gastric and oesophageal squamous cell carcinomas (SCC), hepatocellular carcinoma (HCC), and gastric cancers [56,57]. CD44 plays an essential role in the modulation of the extracellular matrix (ECM), cell migration, and differentiation by transmembrane glycoproteins and has been linked to tumour growth and survival [58]. It has been reported to be up-regulated in pancreatic, colon, prostate, head and neck, and other cancers.

Integrins also belong to the CD antigens group. They mediate adhesion between the cell and its extracellular matrix (ECM). The literature shows that integrins serve multifunctional roles in cancer development including initiation, proliferation, cell migration, intravasation into the vascular system, etc. [59]. CSC-specific integrins have been reported for many cancers, including lung, prostate, colon, glioblastoma, pancreas, and breast cancers.

EpCAM, or an epithelial cell adhesion molecule, is expressed on the basolateral surfaces of most epithelia cells, and overexpression is a marker for most epithelial cancers. It has also been confirmed as a stemness biomarker in HCC [60]. Yamashita et al. [61] performed gene expression profiling and immunohistochemistry analyses of 235 tumour samples and concluded that HCC growth and invasiveness was dictated by a subset of EpCAM(+) cells that displayed cancer stem cell-like traits including the abilities to self-renew and differentiate [61].

Epidermal growth factor receptor (EGFR) is also a transmembrane glycoprotein, involved in a number of cellular processes including proliferation, differentiation, apoptosis, and metabolism [62]. It has been shown that the deregulation of the EGFR pathway leads to the acquisition of stem-like properties in non-small-cell lung cancer [63].

Keratin 19 (K19), a hepatic progenitor cell marker, has been also identified in HCC and successfully used in PET imaging using 18F-FDG [64].

Another protein from the CD family is CD54, also known as ICAM-1, as it is encoded by the ICAM-1 gene. In cancer, ICAM-1 was reported to promote cancer cell migration, invasion, as well as the increase in mesenchymal marker expression. It has been identified as a potential CSC biomarker for esophageal squamous cell carcinoma [65]. It has also been used as a biomarker with the PET and SPECT imaging of pancreatic cancer xenografts while monitoring the xenograft response to radiation therapy, and it was concluded to have biomarker properties to predict the radiation response. However, this use did not necessarily involve the imaging of the CSCs themselves [66].

While many other CSC markers have been extensively discussed in the literature (e.g., [34,47,67]), only a selection of those that have been used in the imaging of CSCs have been mentioned here. The next section of the manuscript will summarise some of the imaging studies reported.

**Table 2:** Selected examples of CSC markers that can be utilised in imaging.

CSC Marker	Cancer Site	References
CD133+	lung, pancreatic, colon, prostate, brain, liver	[50,68]
ABCG2high	lung cancer	[69,70]
CD44+	pancreas, colon, prostate, head and neck	[71,72,73,74]
EpCAM+	pancreas, colon, liver	[60,75]
CD24+	pancreas, colon	[71]
CD138−	multiple myeloma	[76]
CD166+	colon	[77]
CD90+	brain	
CD49f+	brain	
CD38	lung	[78]
CD90-CD117	leukemia	[78]
CD19	ALL, NHL, MCL	
EGRF	glioblastoma, breast, and lung cancers, as well as renal, pancreatic, ovarian, and head and neck cancers	[79]
Integrins	lung, prostate, colon, glioblastoma, pancreas, breast	[80]
Keratin 19	hepatocellular carcinoma	[64]

An important category of CSCs is represented by circulating tumour cells, that, once identified, can offer additional information regarding certain properties of the primary tumour, such as aggressiveness, proliferation, and metastatic potential. CTC identification is usually conducted via immune-based detection reliant on the selective binding of antibodies on cell surface antigens [81]. One of the most common CTC-specific surface molecules is the EpCAM, which is present in most solid cancers but not expressed by peripheral blood cells, thus rendering it a good candidate for the differential identification of cells present in the blood stream [82].

Nevertheless, the employment of EpCAM for CTC identification is limited by cells that express low levels of this surface molecule; thus, EpCAM-independent markers are also required for an accurate quantification. To overcome this shortcoming, other CTC-specific marker genes were identified, such as tumour-specific antigen 9 (TSA-9); cytokeratin (CK) 18, 19, 20; pre-progastrin-releasing peptide (Pre-proGRP) [83,84]; as well as tumour immune markers including EGFR, HER2, and prostate-specific antigen (PSA) [85]. The combination of different antibodies specific for surface proteins and extracellular matrix components is another strategy applied to cases with low EpCAM expression [81]. As such, a number of cell surface-specific antibodies were employed in various cell lines to optimise the identification of CTCs: breast cancer (anti-Trop2, -CD49f, -CD146, -CK8, -c-Met, -CD44, -CD47, -AQP5, -ADAM8, -TEM8) [81]; non-small cell lung cancer (ALDH1, EGFR, Met, HER3) [86]; head and neck cancer (cytokeratin, EGFR, vimentin, N-cadherin, CD44) [87].

Due to the heterogenous nature of CTCs and their tumour specificity, candidate markers are also tumour-specific, similarly to cancer stem cells identified in solid tumours. Research results hint towards the simultaneous use of several cell surface-specific antibodies for more reliable detection and quantification of circulating tumour cells.

## 4. In Vivo Molecular Imaging of Cancer Stem Cells

The imaging of cancer stem cells has been an active area of research in the last two decades. The successful identification and isolation of CSCs under in vitro and ex vivo conditions, employing techniques such as flow cytometry [88] and magnetic-activated cell sorting [89], have largely been reported [58,90]. Of great value to CSC imaging, progress is also being made in the identification of CSC prognosis using CSC and cell line-specific surface properties (e.g., immunochemistry-based molecules utilised to target CSC overexpressed antigens), CSC-specific gene expression (e.g., aldehyde dehydrogenase (ALDH) activity), and CSC-unique surrounding environments (e.g., nanoparticle-based probes) [58,90,91]. Although in vitro techniques, using appropriate markers, could isolate and quantify CSCs, the behaviour and phenotypical progression of these cells that have been isolated from their natural niche remain unknown. In addition, the invasiveness of these techniques makes their application in translational medicine and treatment follow-up impractical. These reasons, along with recent advances in in vivo imaging modalities in terms of resolution and functional imaging improvements, as well as the evolution in drug delivery systems through nanotechnology that has overcome current limitations including the off-target effect, rapid clearance from the body, and others [92] have lent support to the rapidly developing research in this area.

The identification and tracking of CSCs in their natural environment are an integral necessity for the translation of CSC molecular biology into clinical application and the development of case-specific therapeutic strategies. In addition, CSC tracking in vivo could reveal information on the fate of CSC post-treatment and further our knowledge of CSC behaviour and phenotypical evolution. Current CSC in vivo imaging techniques are categorised into studying exogenous and endogenous CSCs. Studying exogenous CSCs involves in vivo flow cytometry using photoacoustic (PA), fluorescence high-resolution optical imaging techniques. The PA method, involving the irradiation of selected vessels using short laser pulses followed by the detection of laser-induced acoustic waves (PA signals) with an ultrasound transducer, is non-radiative imaging that takes advantage of the high sensitivity of laser technology and high spatial resolution and depth of penetration of the ultrasound methods. The application of PA for in vivo CSC imaging for several cancer types has been explored, and it remains an area of active development [93,94].

The most common optical imaging techniques are fluorescent imaging (FI), bioluminescence imaging (BI), and quantum dots (QD). FI offers a high-resolution visualisation of small numbers of cells, but the method is invasive [95,96]. However, due to tissue attenuation and refraction, this method is often used in ex vivo, and hence is invasive [97]. BI provides the advantage of being non-invasive; however, the method demonstrates low spatial resolution and insufficient tissue penetration efficiency of the light [98,99]. A recent development in the BI technique has explored the use of near infrared light NIR I (700–900 nm) and NIR II (1000–1700 nm) instead of visible light to improve tissue penetration and reduce the scatter artefact at intersections. The ability of BI with the NIR-II window to extract tissue information in the range of a centimetre at the micro-level resolution within the first few millimetres’ depth has been reported [100]. QDs are semiconductor nanocrystals with fluorescence features and can detect a resolution of as fine as a fraction of µm [101,102]. QDs with a NIR window provide the highest penetration among the above-mentioned optical imaging methods [103]. Optical and cytometric imaging is not in the scope of this overview and has been largely reported in the literature, e.g., [58,90,97].

In this review, we focus on the application of functional MRI and nuclear medicine imaging techniques, PET and SPECT, for the identification and tracking of endogenous CSCs. The application of MRI has long expanded from providing anatomical information to a functional imaging method with the possibility of visualising dynamic processes and certain activities. The visualisation of different structures or effects is possible using optimised imaging sequences, developed by modulating translational and longitudinal relaxation times. Frequently, the contrast in an image is enhanced using a suitable contrast agent, or more recently, nanomaterial smart probes, which are of particular interest for CSC imaging. Smart or activatable probes function on an ON/OFF basis, that is, the MRI signal is weak in the absence of a certain external stimulus and once triggered the probe is activated, resulting in a detectable change in the contrast, evidencing the presence of a certain enzyme or other dynamic features. A review has been published on the current status of developments of smart/bioresponsive probes in MRI [104].

Generally in MRI, paramagnetic gadolinium-based agents or magnetic nanoparticles are conjugated with antibodies related to a cancer-specific marker (Table 3), e.g., CD44, CD133, and ALDH [105], to pinpoint cells overexpressing the marker [3]. Human hepatic stem cells transplanted into mice were detected and monitored using magnetic iron microbeads conjugated with HEA-125 antibodies that target epithelial cell adhesion molecule (EpCAM) antigens expressed by liver CSC [106]. Choi et al. [107] explored the efficiency of ferritin-based MRI for the in vivo imaging of human breast CSCs (BCSC). This was an animal study whereby BCSCs transduced with the MRI reporter gene, i.e., human ferritin heavy chain (FDH), were transplanted into mice for tracking the CSC growth and response to chemotherapy. Subsequently, a histochemical analysis of excised tissues was performed for CD44 and CD24 markers to verify the MRI findings. To reduce the toxicity from the overexpression of FDH, a doxycycline-triggered genetic reporter system was designed and the feasibility of safely tracking CSCs using FDH using this system was confirmed [108]. Recently, the same group explored the possibility of imaging the malignant transformation of C6 glioma cells [109]. The MRI reporter gene FDH was combined with the tumour-specific promotor, and gene progression elevated gene-3 (PEG3) to construct the lentiviral (LV) vector PEG3@LV@FDH. Although the system succeeded in detecting the CSC malignant transformation in vitro, only CSC detection was observed in vivo. This observation further supports the current suspicion that in vitro experiments may not truly represent the behaviour of CSC in their natural niche.

Cotti et al. [110] demonstrated that breast CSCs exhibit enhanced expression of the L-Ferritin receptors compared to differentiated cells and exploited this feature for the in vivo targeting of CSCs. A contrast-enhanced MRI was used employing the Gd-HPDO3A contrast agent loaded with Apoferritin and Curcumin, which is an anticancer therapeutic agent. Although the low sensitivity of MRI hindered the detection of CSCs that constitute a small proportion of the tumour population, the specificity of Apoferritin was proven, potentially for application in PET.

In vivo imaging using nanoparticle drug delivery systems has demonstrated potential and become a developing area, with studies addressing its current caveats resulting in reduced efficiency. Sun et al. [111] demonstrated that superparamagnetic iron oxide nanoparticles (SPION) conjugated to an extra domain-B of fibronectin (EDB-FN)-specific peptide ligand (APT_EDB_-SPION) could detect EDB-FN (breast CSC biomarker) overexpressing cells. The follow-up work of this group demonstrated that APT_EDB_-TCL-SPIONs loaded with doxorubicin (Dox) can selectively target breast CSCs in vivo using MRI [112]. Al Faraj et al. reported the improved targeting of breast cancer CSC using single-walled carbon nanotubes (SWCN) conjugated with CD44 antibodies and tagged with SPIONs and ^67^Ga for MRI and SPECT methods, respectively [92]. Zhu et al. designed a T1/T2 dual-mode MRI using Fe_3_O_4_@PMn-aptamer nanoparticles as an imaging agent to monitor tumour hypoxia and subsequently CSC-rich areas [113]. The authors based their study on the fact that tumour hypoxia leads to the activation of HIF-1α aptamer which plays an important role in CSC maintenance. Although this study successfully detected areas of enhancement and darkness in T1 and T2, respectively, the specificity for CSC targeting is debatable considering the very small number of CSC cells. An immunochemistry analysis of colorectal cancer lesions indicated that the expression of CSC markers, CD44 and CD166, were strong in the regions that exhibited a high glutamine uptake on in vivo MRI imaging [114]. The authors exploited the previous finding of their group [115] correlating CSC suppression and glutamine transporter (ASCT2) inhibition to propose a CSC-targeting therapy using ASCT2 distributions on MRI images.

With a therapeutic intent, Tang et al. proposed targeting CSCs through their signalling pathway using the hybrid nano delivery system Fe_3_O_4_@PPr (polypyrroles)@HA (Hyaluronic acid) loaded with DAPT, which is a Notch signalling pathway inhibitor [116]. They successfully demonstrated the efficiency of the proposed method for CSC detectability in vivo (via DAPT which is an indication of CD44^+^ signature) and eradication.

**Table 3:** Molecular imaging methods developed for cancer stem cell imaging in vivo.

Molecular Imaging	Cell Line	Imaging Agent	CSC Biomarker	Type	Reference
MRI	Liver	HEA125@magnetic iron microbeads	EpCAM	Mice	[106]
MRI	Human breast	FDH heavy chain	CD44^+^/CD24^−^	Mice	[107]
MRI	Neuroblastoma	LV@Tet@FDH	Iron	Mice	[108]
MRI	C6 Glioma	PEG3@LV@FDH	Iron	Mice	[109]
MRI	Human breast	Apoferittin	Iron	Mice	[110]
MRI	Human breast	APT_EDB_@SPION	EDB-FN	Mice	[111]
MRI	Human breast	Dox@APT_EDB_@SPION	EDB-FN	Mice	[112]
Dual MRI	Pancreas	Fe_3_O_4_@PMn NPs	HIF-1α aptamer	Mice	[113]
MRI	Human breast	SWCN + SPION	CD44	Murine	[92]
SPECT	Human breast	^67^Ga	CD44	Murine	[92]
MRI	Human and murine breast	Fe_3_O_4_@PPr@HA + DAPT	CD44	Mice	[116]
MRI	Human colon	ASCT2	CD44, CD166	Mice	[114]
PET	Mice colon	^64^Cu-ATSM	CD133	Mice	[117,118]
PET	Human CT26 colon	^125^I-ANC9C5	CD133	Mice	[119]
PET	Mice CT26 colon	^125^I-NIS	CD133	Mice	[120]
PET	Human glioma U251 and GBM NCH421k	^64^Cu-NOTA-AC133 mAb	CD133	Mice	[68]
PET	Human GBM U87MG	^64^Cu-NOTA-YY146	CD146	Mice	[121]
SPECT	Human GBM U87MG	^111^I- MSN-DOTA	-	Mice	[122]
PET	TNBC	^89^Zr@CS-GA-MLP	CD44	Mice	[123]
PET	Human breast	^18^F-FDG	CSC metabolic phenotype	Human retrospective study	[124]
SPECT/NIR	Human colon HCT116	^99^Tc-TEx-Cy7	-	Mice	[125]
PET/MRI	MSC	^18^F- Fe_3_O_4_@Al(OH)_3_	-	Mice	[126]

Nuclear medicine imaging, including PET and SPECT, offers highly sensitive and non-invasive techniques that detect high-energy γ-rays emitted from radiopharmaceutical agents administered to a patient before the image acquisition. A previous review of this group reported on the then-current status of the development of various radiotracers developed for different tumour characteristics and molecular pathways including stemness [127]. A number of these radioligands have successfully been implemented for the identification and tracking of CSCs in vivo (Table 3).

Yoshii et al. used the ^64^Cu-diacetyl-bis (N_4_methylthiosemicarbazone) (^64^Cu-ATSM) PET radiotracer for in vivo imaging of mice bearing colon carcinoma (colon-26) [118]. It was demonstrated that ^64^Cu-ATSM was localised in regions with a high density of CD133^+^ cells, a protein known to be overexpressed by various animal and human CSCs. The next study of the group showed the therapeutic impact of this radiotracer, whereby, as compared to the control group, a reduction in the percentage of CD133^+^ cells was observed [117]. The PET radiotracer ^125^I labelled by the anti-CD133 antibody ANC9C5 (^125^I-ANC9C5) showed a higher accumulation in overexpressing CD133 cells in colon cancer (HCT166)-bearing mice [119]. In the context of the longitudinal monitoring of CSCs and understanding the impact of the CSC niche on the fate of these cells, in vivo PET imaging strategies have been developed based on reporter gene systems that produce signals only in viable cells [127,128]. Park et al. explored the efficiency of in vivo PET using the sodium/iodide symporter (NIS) reporter gene radiolabelled by ^124^I to identify and monitor CSCs in living mice infected by mice CT26 colon CSC (confirmed for the CD133 expression in a Western blot analysis) [120]. Areas of CSCs were clearly visualised due to enhanced uptake. In addition, this reporter imaging was able to identify microenvironmental elements affecting the fate of implanted CSCs at early stages.

The AC133 epitope of CD133 antigen is known as a marker of aggressive brain CSCs such as Glioblastoma Multiforme (GBM) and various pediatric brain malignancies [129]. Gaedicke’s group [68] developed an antibody-mediated PET radiotracer and successfully detected and monitored AC133^+^ GBM (NCH421k) and U251 glioma CSCs in mice. The AC133 monoclonal IgG antibody (mAb), which identifies the AC133 epitope on CD133-overexpressing cells and hence CSCs, was conjugated with chelator NOTA and radiolabelled by ^64^Cu to make the ^64^Cu-NOTA-AC133 mAb radiotracer. Similarly, the ^64^Cu-NOTA-YY146 PET radiotracer was developed to target the CD146 marker known to be overexpressed in aggressive brain CSC via YY146, which is an anti-CD146 antibody [121]. This PET radiotracer not only was able to detect CSCs in mice bearing GBM U87MG xenografts in vivo but also showed a therapeutic effect on CSCs. These observations suggested the clinical applicability of ^64^Cu-NOTA-YY146 in patient stratification and targeted therapies. Cheng et al. [122] developed a strategy for the in vivo tracking of migrating neural stem cells (NSCs) toward glioblastoma via SPECT using a mesoporous silica nanoparticle (MSN)-^111^ in probe. SPECT data corroborate with the bioluminescence live imaging and histological analyses.

Triple-negative breast cancer (TNBC) presents clinically with inherently aggressive features with high rates of metastasis and recurrence [130]. Human TNBC CSC clusters confirmed for elevated CD44 expression were quantitively traced in vivo using an antibody-mediated PET strategy [123]. The PET radiotracer ^89^Zr@CS-GA-MLP was fabricated using multifunctional liposomes (MLPs) surface-decorated with chitosan (CS), loaded with gambogic acid (GA), and radiolabelled with ^89^Zr. The results of this study demonstrated the capacity of ^89^Zr@CS-GA-MLP for PET-image-guided, CD44-targeted TNBC therapy in the future. In a different approach, a recent study explored the possibility of breast CSC differentiation from differentiated cancer cells using the CSC’s distinct metabolic phenotype known as the Warburg effect [124]. The Warburg effect [131] indicates that enhanced glycolysis is the marker of CSC metabolism, which can potentially be measured by ^18^F-FDG PET. ^18^F-FDG PET/CT scans of 129 patients with stage II and III breast cancer who underwent chemotherapy were retrospectively analysed. The intratumoural heterogeneity in terms of glucose metabolism was quantified with high standardised uptake values (SUVs) hypothesised to be associated with CSCs. The association between the metabolic tumour volume for CSC (MTV_csc_) and complete response (pCR) and disease-free survival (DFS) was assessed. It was concluded that MTV_csc_ was predictive of DFS and pCR in TNBC/HER2^+^ subtypes, and therefore MTV_csc_ can be used as guidance for treatment optimisation.

Multimodal imaging approaches have recently been considered to overcome the limitations of individual modalities. For example, simultaneous PET and MRI acquisitions could produce images with high contrast resolution (MRI) and functional sensitivity (PET) for long-term in vivo tracking using double agent contrasts. The potential of ^18^F-labelled Fe_3_O_4_@Al(OH)_3_ nanoparticles for the simultaneous PET/MR imaging for short- and long-term tracking of mesenchymal stem cells (MSCs) transplanted into mice was demonstrated in the study of Belderbos [126]. SPECT/NIR is another promising method as SPECT imaging provides excellent penetration and sensitivity, while NIR imaging offers a high temporal and spatial resolution. Jing et al. [125] demonstrated the efficacy of dual SPECT and NIRF I (650–1000 nm) imaging using a tumour cell-derived exosome nanoprobe (TEx-Cy7) radiolabelled with ^99m^Tc for tracking the adipose stem cells (ASCs) of human colon cancer injected into mice.

### Imaging of Circulating Tumour Cells

As mentioned in the previous sections, circulating tumour cells represent a phenotypically and functionally diverse population of cells originating from the primary tumour that are able to develop distant metastases. As their pathway to distant anatomic sites is through the blood stream, the most common approach to identify CTCs is via liquid biopsies. However, CTC isolation and detection pose certain challenges as the employed technique must have high sensitivity and specificity to truthfully identify these cells. Consequently, CTC-specific techniques should hold the following requirements to differentiate CTCs from other blood cells: to be negative for the hematopoietic cell marker CD45; to be positive for cytokeratin, the structural protein expressed by epithelial cells; and to be positive for EpCAM, the epithelial cell adhesion molecule with the role of surface marker [132]. The most commonly used techniques to detect CTCs from the peripheral blood are the CellSearch system (the gold standard), the CTC chips, and the Isolation by Size of Epithelial Tumour Cells (ISET) filter device, with the first two employing EpCAM for CTC identification. There are, however, reports suggesting that the CellSearch system is likely to underestimate the number of cells that express EpCAM (due to the down-regulation of EpCAM expression in CTCs that undergo EMT); therefore, alternative strategies that are independent of this marker are recommended [133]. The ISET filter device exploits physical features, such as size, that differentiate cancer cells from normal hematopoietic cells, showing better potential for CTC quantification and monitoring than EpCAM-based techniques [133].

While CSC imaging within solid tumours via PET, SPECT, or MRI offer information on functional properties at a given timepoint, the identification of CTCs in similar conditions, i.e., at a single timepoint, might not offer relevant data, unless continuous monitoring of CTCs is conducted via subsequent liquid biopsies to obtain a dynamic picture of the metastatic progress [134]. In view of this, circulating tumour cells could serve as direct markers or surrogates for clinical outcome prediction and treatment response monitoring, while also accelerating the development of CSC-specific therapies.

## 5. Challenges and Future Prospects

Cancer stem cells came to the research community’s attention much later than other tumour properties that encumber the optimal treatment outcome, such as hypoxia, intrinsic radioresistance, angiogenesis, accelerated proliferation, etc. However, there is already clinical evidence supporting the need for CSC identification towards efficient targeting and eradication. Challenges still exist, as there are several biological, treatment-, and host-related factors that require a better understanding before an efficient translation from bench to bedside comes into effect. Challenges to be overcome in the field of CSCs related to the current review include, but are not limited to:

1. Biological/treatment-related issues:
  (a) To identify and characterise the various CSC phenotypes for a more personalised approach and patient stratification.
  (b) To elucidate the role of quiescent CSCs in tumour progression and dissemination; should they be targeted in their dormant phase or triggered into the cell cycle?
  (c) To obstruct DNA repair pathways in CSCs to improve cellular radiosensitisation.
  (d) To identify the need for interference with cellular plasticity and its pathways for keeping the CSC subpopulation to a minimum.
  (e) To clarify the role and the need for cell-differentiating agents in tumour sensitisation.
  (f) To further investigate the impact of fractionated radiotherapy on CSC dynamics.
2. CSC identification/marker-related issues:
  (a) To develop tumour-specific CSC markers with high specificity for in vivo imaging.
  (b) To reliably quantify the in vivo CSC population based on specific markers.
  (c) To identify phenotype-specific CSCs in view of the differential response to therapy.
  (d) To clinically evaluate other than through cell surface markers (which are not always uniformly useful in identifying CSCs) for a more reliable patient stratification.
  (e) To identify and employ markers that interconnect with the physiological function of CSCs, providing more accurate detection.
3. In vivo imaging-related issues:
  (a) To perfect in vivo imaging of endogenous CSCs. Although single-cell resolution MRI imaging has been reported for small animals in exogenous models, in vivo imaging of endogenous CSCs at the cell level is still a developing area.
  (b) To develop high sensitivity and specificity of MRI- and PET-compatible agents that target CSCs. Advances in technology and radiotracer fabrications, along with high functional and molecular sensitivity, have made PET’s potential equal to MRI.
  (c) To develop hybrid imaging techniques for CSCs. Combined modalities have shown promise, although not yet at the proof-of-principle capacity, to overcome several limitations of individual modes.
  (d) To encourage the translation of in vivo CSC imaging from animal studies to humans. The number of human studies on the functional imaging of CSCs is still scarce.
  (e) To improve the detection resolution of non-invasive modalities at the cellular level. Currently, in vivo imaging is able to identify regions/clusters containing CSCs; nevertheless, the translation of CSC biology to clinical application relies on the identification of individual CSCs due to their scarcity.

Solutions to the above-highlighted challenges could advance our current knowledge of CSCs and offer new and improved avenues for more accurate identification and targeting of this subpopulation of cancer cells, and thus lay the foundation for future translational studies.

## Data Availability

All the data is cited in this article.
